# Vibriocidal Antibody Responses to a Bivalent Killed Whole-Cell Oral Cholera Vaccine in a Phase III Trial in Kolkata, India

**DOI:** 10.1371/journal.pone.0096499

**Published:** 2014-05-06

**Authors:** Suman Kanungo, Anna Lena Lopez, Mohammad Ali, Byomkesh Manna, Deok Ryon Kim, Tanmay Mahapatra, Jan Holmgren, Mandeep S. Dhingra, Thomas F. Weirzba, G. Balakrish Nair, Sujit K. Bhattacharya, John D. Clemens, Dipika Sur

**Affiliations:** 1 Department of Epidemiology, National Institute of Cholera and Enteric Diseases, Kolkata, West Bengal, India; 2 University of the Philippines, National Institutes of Health, Manila, Philippines; 3 International Vaccine Institute, SNU Research Park, Nakseongdae-dong, Gwanak-gu, Seoul, Korea; 4 University of Gothenburg, Gothenburg, Sweden; 5 Shantha Biotechnics Limited, Hyderabad, Andhra Pradesh, India; 6 Executive Director, Translational Health Science and Technology Institute, Gurgaon, Haryana, India; 7 Senior Scientist Platinum Jubilee Fellow, The National Academy of Sciences, Allahabad, India; 8 Executive Director, International Centre for Diarrheal Disease Research, Dhaka, Bangladesh; University of Melbourne, Australia

## Abstract

**Background:**

During the development of a vaccine, identification of the correlates of protection is of paramount importance for establishing an objective criterion for the protective performance of the vaccine. However, the ascertainment of correlates of immunity conferred by any vaccine is a difficult task.

**Methods:**

While conducting a phase three double-blind, cluster-randomized, placebo-controlled trial of a bivalent killed whole-cell oral cholera vaccine in Kolkata, we evaluated the immunogenicity of the vaccine in a subset of participants. Randomly chosen participants (recipients of vaccine or placebo) were invited to provide blood samples at baseline, 14 days after the second dose and one year after the first dose. At these time points, serum geometric mean titers (GMT) of vibriocidal antibodies and seroconversion rates for vaccine and placebo arms were calculated and compared across the age strata (1 to 5 years, 5 to 15 years and more than 15 years) as well as for all age groups.

**Results:**

Out of 137 subjects included in analysis, 69 were vaccinees and 68 received placebo. There were 5•7 and 5•8 geometric mean fold (GMF) rises in titers to *Vibrio cholerae* Inaba and Ogawa, respectively at 14 days after the second dose, with 57% and 61% of vaccinees showing a four-fold or greater titer rise, respectively. After one year, the titers to Inaba and Ogawa remained 1•7 and 2•8 fold higher, respectively, compared to baseline. Serum vibriocidal antibody response to *V. cholerae* O139 was much lower than that to Inaba or Ogawa. No significant differences in the GMF-rises were observed among the age groups.

**Conclusions:**

The reformulated oral cholera vaccine induced a statistically significant anti-O1 Inaba and O1 Ogawa vibriocidal antibody response 14 days after vaccination, which although declined after one year remained significantly higher than baseline. Despite this decline, the vaccine remained protective five years after vaccination.

## Introduction

The past decade has seen an increase in the number of cholera outbreaks worldwide [1]. An inexpensive, killed oral cholera vaccine (OCV) was produced in Vietnam in 1997 following technology transfer from Sweden. Various generations of the vaccine were found to be safe and protective [2], [3]. The vaccine was reformulated to comply with WHO recommendations [4] and current Good Manufacturing Practices while the vaccine production technology was transferred to a manufacturer in India (Shantha Biotechnics Limited), where the national regulatory authority was WHO recognized.

Prior to the transfer of the technology to India, immunogenicity studies were first conducted in adults in SonLa, Vietnam (a cholera non-endemic area) [5] and in children and adults in Kolkata, India (a cholera endemic region) [5], [6]. In SonLa, 90% of the vaccine recipients, aged 18–48 years, developed ≥ four-fold rise in vibriocidal antibodies to *V. cholerae* O1 Inaba, and there was a 26.8-fold rise in the geometric mean titers (GMT) 14 days after the second dose suggesting the reformulated vaccine was highly immunogenic. Overall geometric mean-fold (GMF) rises in serum antibodies were lower in Kolkata (4.5-fold in adults and 12.6-fold in children) than that in SonLa (26.8-fold) where only adults participated. The lower GMF rises seen in Kolkata were explained by higher levels of pre-existing vibriocidal antibody titers in Kolkata compared to that seen in SonLa [5]–[8].

Oral cholera vaccines stimulate anti-LPS secretory IgA responses similar to infection itself, but it is impractical to obtain intestinal immune responses in large-scale clinical trials [9]. Currently, no correlate of protection exists for oral cholera vaccines, however serum vibriocidal antibody responses that appear following the ingestion of antigens have been used as indicators for development of potential intestinal immunity that endures long after the serum vibriocidal antibody titres have returned to baseline levels [4]. The results of the studies conducted in SonLa and Kolkata indicated that the vaccine was immunogenic and likely protective against cholera. During the Phase III cluster-randomized, double blind, placebo-controlled trial of the reformulated OCV conducted in Kolkata to evaluate the efficacy of the vaccine [10], immunogenicity was assessed in a small subset of individuals at 14 days and 1 year after vaccination.

## Methods

### Ethics statement

The study protocol was approved by the Drugs Controller General of India, the Ethics Committee of the National Institute of Cholera and Enteric Diseases, the Health Ministry Screening Committee of India and the International Vaccine Institute Institutional Review Board.

Written informed consent was obtained from residents older than 18 years and from the guardians of residents aged 1 to 17 years. Written assent was obtained from residents aged 12 to 17 years. Additional consent and assent forms were obtained from participants included in the immunogenicity subset. An independent data and safety monitoring board reviewed the study protocol, assessed serious adverse events, and approved freezing of data and the analytical plan prior to starting the analysis.

The trial was registered at ClinicalTrials.gov number, NCT00289224.

### The vaccine

Each dose of the modified killed whole cell vaccine contained 600 ELISA units (EU) of lipopolysaccharide (LPS) of formalin-killed *Vibrio cholerae* O1 El Tor Inaba (strain Phil 6973), 300 EU of LPS of heat-killed *V. cholerae* O1 classical Ogawa (strain Cairo 50), 300 EU of LPS of formalin-killed *V. cholerae* O1 classical Ogawa (strain Cairo 50), 300 EU of LPS of heat-killed *V. cholerae* O1 classical Inaba (strain Cairo 48) and 600 EU of LPS of formalin-killed *V. cholerae* O139 (strain 4260B). Identical vials containing heat-killed *Escherichia coli* K12 were used as placebo. Vaccine and placebo were stored at temperature between 2 to 8°C until dosing. Vaccine was presented in single dose vials labeled with one of four letter codes, two for vaccine and two for placebo.

### The trial

The Phase III trial was conducted in a cholera-endemic area in Kolkata encompassing a population of about 109,000. Details of the study site and study procedures were previously reported [11], [12]. Briefly, residents aged one year and older who were not pregnant were invited to participate. Eligible residents (107,774) were cluster-randomized (3,933 clusters), using dwellings as clusters and pre-assigned to receive two-dose regimens of either the oral cholera vaccine (OCV), or oral placebo so that subjects residing in the same dwelling received identical intervention. Enrollment and administration of the pre-assigned agents were performed after acquisition of written informed consent by dosing teams in vaccination centers serving the population.

### Subjects and sampling

For the immunogenicity subset, from the list of 107,774 eligible residents of the study area, by stratified random sampling, a list of residents was generated from which we planned to enrol 300 subjects based on: allocated agent (vaccine or placebo) and age group (less than 5 years, 5 to 15 years and over 15 years of age).

Assuming a 5 % background rate of response in the placebo group after the second dose and the true rate of vibriocidal responses in the vaccine group being 25%, at p<0·05 (one tailed), to have 80% power with an 1∶1 allocation of subjects in the vaccine and placebo, 46 subjects per arm per age group (1 to 5 years, 5 to15 years and more than 15 years) were required. Thus approximately a total of 300 subjects were planned to be recruited for the age-stratified analyses, accounting for drop-outs.

### Randomization and masking

A statistician who was otherwise not involved in the study prepared the randomization list. Study participants and the investigators were blinded to the study agent (whether vaccine or placebo) applied to each individual. Technicians blinded to the study agent received by the subjects, performed the assays.

### Study procedures and definitions

5 ml of blood were obtained from the selected subjects at baseline (prior to administration of the study agent), 14 days after the second dose and one year after the first dose. The microtiter technique was used to detect serum vibriocidal antibodies to *V. cholerae* O1 El Tor Inaba strain (T19479) and El Tor Ogawa strain (X25049) [13]. For the serum vibriocidal antibodies to *V. cholerae* O139, a modified microtiter assay was performed at the University of Gothenburg [6], [14]. Two-fold serial dilutions of pre- and post vaccination sera were performed in duplicates, and the mean of the two determinations was the final titer. The assay was repeated if a ≥ two-fold difference was noted between the results of the duplicate tests. Initial serum dilutions for testing were 1∶2.5 for *V. cholerae* O1 and 1∶10 for *V. cholerae* O139, respectively. Vibriocidal titers <2·5 for *V. cholerae* O1 and <10 for *V. cholerae* O139 were considered as 1·25 and 5, respectively, for statistical analyses. Seroconversion was defined as a ≥ four-fold increase in titer of serum vibriocidal antibodies between baseline and post-second dose blood collections. For samples with limited serum volume, the following testing priority was followed: O1 Inaba, O139 and O1 Ogawa.

### Data analysis

The percentages of subjects in the vaccine and placebo groups who seroconverted from baseline to 14 days after the second dose and from baseline to one year after the first dose were calculated and compared among the two dose recipients. Serum vibriocidal titers and fold–rises were logarithmically transformed prior to statistical analyses. Comparison of the GMT and GMF rises between vaccine and placebo groups at baseline, 14 days after the second dose and at one year after the first dose were performed. The Student’s t-test, Welch’s t-test or Wilcoxon rank sum test was used for continuous data (GMT) depending on whether the variance was equal or not and depending on the distribution of data. For categorical data, we used the chi-square or the Fisher’s exact test, if a cell count was sparse.

Comparison of the GMF rise among different age groups was performed using one-way ANOVA. We also derived simulated p-value using simanova implemented in STATA, which was more robust to violation of the homogeneity of variance assumption. Bartlett’s test for equal variance was employed to evaluate homogeneity of the data among age groups. If the test yielded homogeneity in the data, then the nominal p-value of the one way ANOVA was accepted, otherwise the simulated p-value. Two tailed tests were conducted for all analyses.

## Results

**Figure 1:**
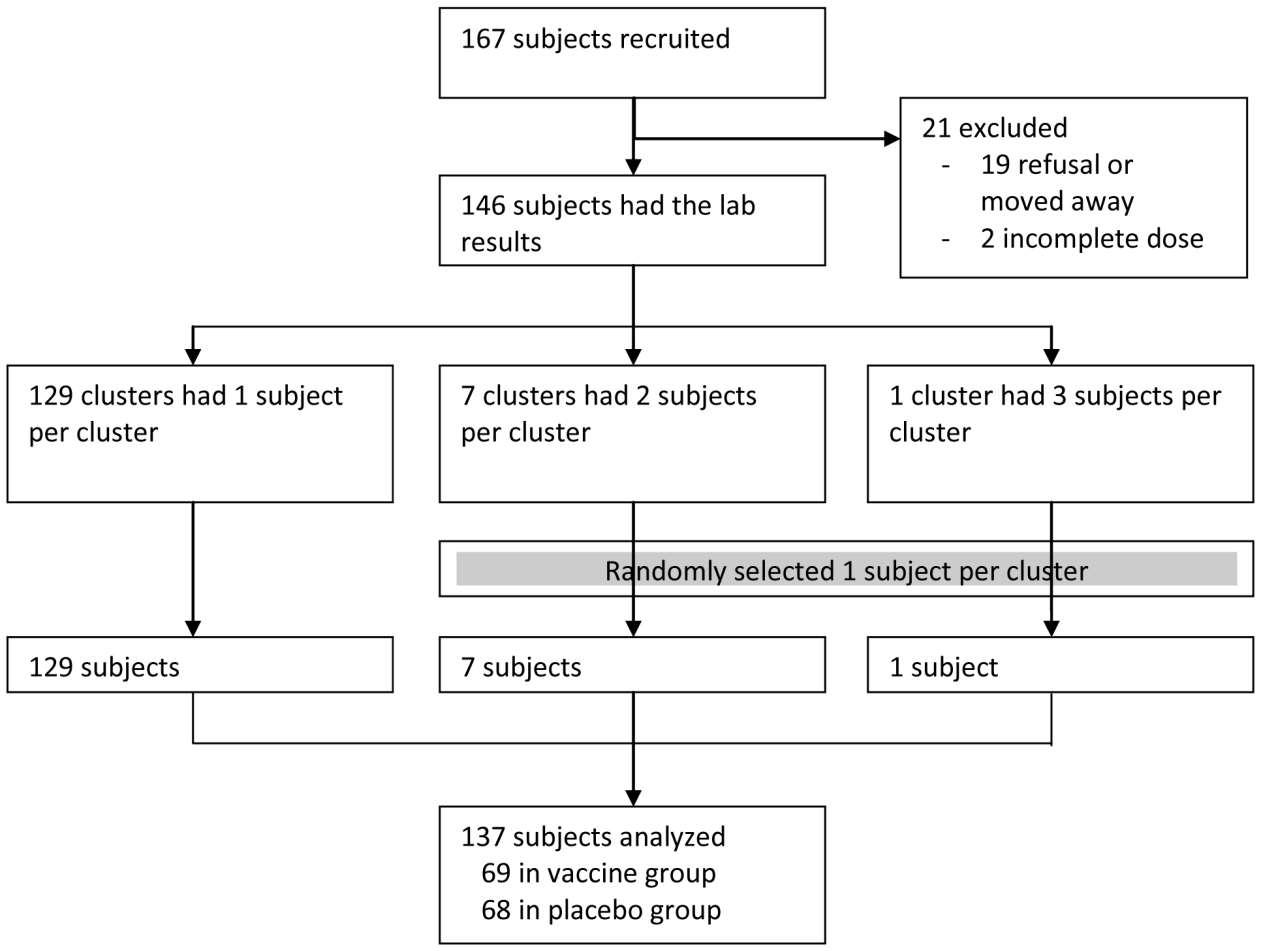
CONOSRT for assembling the subjects.

Among 300 selected subjects, 167 received the first dose of either vaccine or placebo. 19 of them either refused to provide required blood sample or migrated out of the study area before completion of blood collection, and two persons did not accept the second dose. Only one subject in a cluster was taken, thus nine subjects were excluded (Figure 1). Finally, 137 subjects (69 in the vaccine and 68 in the placebo arm) who received both doses and bled at least twice (at baseline and 14 days after the second dose) were included in the analysis. One year after the first dose, there were 19% drop-outs in the vaccine group, and 12% in the placebo group (Figure 1 and Table 1).

**Table 1:** Serum vibriocidal antibody titers to *V. cholerae* O1 Inaba among vaccine and placebo recipients.

	Vaccine			Placebo		
	Baseline	14 days after dose 2	1 year after dose 1	Baseline	14 days after dose 2	1 year after dose 1
**Inaba**	**(n = 69)**	**(n = 69)**	**(n = 56)**	**(n = 68)**	**(n = 68)**	**(n = 60)**
GMT^a^	90.2	518.3	226.3	48.5	55.4	54.6
GMF-rise^b^		5.7	1.7		1.1	1.2
p value^c^		<0.001	0.001			
No (%) seroconverted^d^		39 (56.5)	15 (26.8)		3 (4.4)	5 (8.3)
p value^e^		<0.001	0.01			
**Ogawa**	**(n = 42)**	**(n = 42)**	**(n = 42)**	**(n = 46)**	**(n = 46)**	**(n = 46)**
GMT^a^	115.0	672.5	320.0	98.8	94.4	106.5
GMF-rise^b^		5.8	2.8		1.0	1.1
p value^c^		<0.001	0.001			
No (%) seroconverted^d^		26 (61.9)	13 (31.0)		1 (2.2)	3 (6.5)
p value^e^		<0.001	0.005			
**O139**	**(n = 55)**	**(n = 55)**	**(n = 55)**	**(n = 55)**	**(n = 55)**	**(n = 55)**
GMT^a^	184.7	319.1	271.1	164.0	168.3	182.2
GMF-rise^b^		1.7	1.5		1.0	1.1
p value^c^		<0.001	0.02			
No (%) seroconverted^d^		8 (14.6)	5 (9.1)		1 (1.8)	3 (5.5)
p value^e^		0.03	0.72			

a GMT is Geometric Mean Titer.

b GMF rise is Geometric Mean Fold rise from baseline to 14 days after dose 2 or from baseline to 1 year after dose 1.

c P value for comparison between vaccine and placebo for GMF rise after controlling for the baseline titre.

d No (%) seroconverted from baseline to baseline to 14 days after dose 2 or from baseline to 1 year after dose 1.

e P value for comparison between vaccine and placebo for % seroconversion after controlling for the baseline titre.

For responses against Inaba, a six-fold GMF rise from baseline was seen 14 days after the second dose, which declined to two-fold after one year. The GMF rises at both the time points were significantly different from baseline (p<.01). Approximately 56% and 27% of the vaccinees developed four-fold rise in titers 14 days after the second dose and one year after the first dose respectively. In contrast only 1% of the placebo recipients developed four-fold rise to Inaba at both the time points. The increase of GMT in the vaccine group compared to that in the placebo group was statistically significant at both time points (p<.001 and p = .01 for 14 days after the second dose and one year after the first dose, respectively). We used 42 pairs of samples for serum vibriocidal tests against the Ogawa serotype and 55 pairs of samples for tests for responses to O139 serogroup amongst vaccinees. The results of the vibriocidal test for Ogawa were similar to that for Inaba (Table 1). However, GMF rises to O139 were much lower than that to Inaba or Ogawa. Titers at one year were lower compared to 14 days after the second dose (Table 1). The seroconversion rate at one year after the first dose declined by ∼50% for all strains while the decline in the seroconversion rate against O139 at one year after the first dose was not statistically significant.

The distribution of samples by age at dosing (1 to <5 years, 5 to <15 years, and 15 years and older) for the vaccine recipients is shown in Table 2. Although the GMF rise to Inaba, Ogawa, and O139 was higher among younger subjects (<15 years old) than that among older subjects at 14 days after the 2^nd^ dose, the rise was not significantly different among the different age groups (p-values for Inaba, Ogawa, and O139 were 0.08, 0.30, and 0.33, respectively).

**Table 2:** Comparison of geometric mean fold rises across age groups among vaccine recipients in Kolkata, India.

	Age* groups (years)	Median age (years)	Baseline		14 days after the second dose				1 year after the first dose			
			N	GMT†	N	GMT†	GMF‡rise	P-value§	N	GMT†	GMF‡ rise	P-value§
Inaba	1−<5	3.5	8	10.0	8	134.5	13·4	0·081	5	11.5	1·5	0·291
	5−<15	9.0	29	85.9	29	640.0	7·4		21	320.0	2·1	
	15+	34.5	32	163.5	32	599.7	3·7		30	291.7	1·6	
Ogawa	1−<5	1.2	3	25.2	3	320.0	12·7	0·298	3	403.2	16	0·391
	5−<15	9.5	16	54.2	16	697.9	12·9		16	226.3	4·2	
	15+	37.0	23	236.7	23	722.0	3·1		23	395.2	1·7	
O139	1−<5	1.3	5	63.2	5	150.3	2·4	0·333	5	79.2	1·2	0·837
	5−<15	8.0	21	216.7	21	404.7	1·9		21	335.1	1·5	
	15+	35.0	29	198.0	29	305.9	1·5		29	287.4	1·5	

*Age at the date of 1^st^ dose.

† GMT refers to Geometric mean titers.

‡ GMF refers to Geometric Mean Fold.

§ The p-value is either nominal from one way ANOVA or simulated using somanova implemented in STATA depending on the results of the Bartlett’s test for equal variance (described in the text).

## Discussion

The results of our study confirmed that the reformulated bivalent oral cholera vaccine was found to remain immunogenic in this current study, which was a part of a large Phase III trial conducted in an endemic setting in the city of Kolkata. The vaccine elicited significant levels of serum vibriocidal antibodies to Inaba, Ogawa and O139, 14 days after the second dose. These findings were comparable to an earlier study conducted in Kolkata [6]. Although serum vibriocidal responses rapidly declined one year after the first dose for Inaba and Ogawa, these remained significantly different from baseline titers. In earlier studies conducted in Bangladesh [15] and Peru [16] with the B-subunit containing whole cell OCV (Dukoral^®^, Crucell), the vibriocidal titers to Inaba and Ogawa declined to near baseline levels, one year after vaccination. However, in our study, although the titers declined substantially, these remained significantly higher when compared to baseline. Earlier studies on live cholera vaccines: CVD 103HgR, CVD 103-HgR2 and CVD 110 detected higher titers on 10th post-immunization day. [17] Thus if sera were obtained earlier than the14th day post second dose, a higher magnitude and fold increase of responses might have been observed.

The absence of cholera cases among the subgroup of participants in this immunogenicity assessment prevented us from correlating serum vibriocidal antibody responses with vaccine protection. It would have been good to have vibriocidal responses measured in children stratified by age (1–2 years and 2–5 years) although it was not possible to do so due to study design and limitations pertaining to sample size. However, despite the decline in vibriocidal antibodies at one year, the vaccine sustained its efficacy for at least five years post-vaccination [10], [11], corroborating earlier evidence that serum vibriocidal antibody responses are not correlates of protection [4]. Since intestinal IgA antibody levels, which differ from the serum vibriocidal antibodies are believed to mediate the protection after vaccination, also decline within approximately one year while the immunological memory for an anamnestic response persists for many years [18] the most plausible explanation for long-lasting protection despite waning antibody titers is a rapid anamnestic response upon re-exposure curtailing the infection before it causes illness [19].

Because there were no O139 cases seen in the field site since the beginning of surveillance, even prior to the start of the Phase III trial, the clinical significance of the lower vibriocidal titers to O139 remains unknown. The role of serum vibriocidal antibody responses to O139 remains debatable [7], [8].

Due to nonparticipation, the required number of subjects (46 in each age group in each arm) for the age-stratified analysis was not attained and is a major limitation of this assessment. Thus we evaluated the differences in serum vibriocidal responses among different age groups using one-way ANOVA. The results of the tests could not identify any significant differences in GMF rises to Inaba, Ogawa, or O139 across different age groups. However, since we did not have adequate power to evaluate such differences, we could not conclude that the age-related differences in serum vibriocidal responses did not exist.

The absence of a serologic correlate of protection for cholera remains a potential hindrance for future vaccine development, as large, expensive efficacy trials may be necessary for their assessment. Furthermore, performance of serum vibriocidal assays is not standardized between different institutions providing results that limit comparisons. Standardized, validated assays based on internationally available reference sera may be necessary for testing currently available vaccines in different settings and future evaluation of the newer vaccines.

A more recent study conducted in Dhaka confirmed that this reformulated oral bivalent cholera vaccine (now available as Shanchol) was safe. It also elicited vibriocidal (mainly LPS-specific IgM) and LPS-specific serum IgA responses [20] among subjects aged one year and above. This paved the way for a mass oral cholera campaign in endemic areas of Dhaka, Bangladesh. This vaccine is now licensed in several Asian and African countries besides India, and also WHO prequalified since 2011.

As the global cholera problem continues, in addition to measures for the improvement of access to safe drinking water, sanitation and hygiene, WHO also recommended the use of oral cholera vaccines to mitigate the impact of cholera in endemic areas [21].
